# Socially Responsible Surgery: Building Recognition and Coalition

**DOI:** 10.3389/fsurg.2017.00011

**Published:** 2017-04-05

**Authors:** Tyler D. Robinson, Thiago M. Oliveira, Theresa R. Timmes, Jacqueline M. Mills, Nichole Starr, Matthew Fleming, Megan Janeway, Diane Haddad, Feroze Sidhwa, Ryan D. Macht, Douglas F. Kauffman, Tracey A. Dechert

**Affiliations:** ^1^Department of Surgery, Boston Medical Center, Boston, MA, USA; ^2^Department of Emergency Medicine, Boston Medical Center, Boston, MA, USA; ^3^Boston University School of Medicine, Boston, MA, USA; ^4^Department of Surgery, University of California, San Francisco, CA, USA; ^5^Department of Surgery, Yale University, New Haven, CT, USA; ^6^Department of Surgery, Vanderbilt University, Nashville, TN, USA

**Keywords:** disparities, socially responsible surgery, social determinants of health, general surgery, trauma surgery

## Abstract

**Importance:**

Socially responsible surgery (SRS) integrates surgery and public health, providing a framework for research, advocacy, education, and clinical practice to address the social barriers of health that decrease surgical access and worsen surgical outcomes in underserved patient populations. These patients face disparities in both health and in health care, which can be effectively addressed by surgeons in collaboration with allied health professionals.

**Objective:**

We reviewed the current state of surgical access and outcomes of underserved populations in American rural communities, American urban communities, and in low- and middle-income countries.

**Evidence review:**

We searched PubMed using standardized search terms and reviewed the reference lists of highly relevant articles. We reviewed the reports of two recent global surgery commissions.

**Conclusion:**

There is an opportunity for scholarship in rural surgery, urban surgery, and global surgery to be unified under the concept of SRS. The burden of surgical disease and the challenges to management demonstrate that achieving optimal health outcomes requires more than excellent perioperative care. Surgeons can and should regularly address the social determinants of health experienced by their patients. Formalized research and training opportunities are needed to meet the growing enthusiasm among surgeons and trainees to develop their practice as socially responsible surgeons.

## Introduction

Socially responsible surgery (SRS) is a field of study and clinical practice that integrates surgery and public health and emphasizes addressing the social determinants of health affecting our patients. SRS unifies academic inquiry in rural surgery, urban surgery, and global surgery by its mission to provide quality surgical care to underserved patient populations. In addition to maintaining high standards of clinical expertise, surgeons practicing SRS skillfully address disparities of care rooted in social, cultural, economic, and geographic factors, thereby improving access to and outcomes from surgical care. These surgeons provide care to individual patients from underserved communities, have impact on populations through systemic interventions, and experience personal and professional growth through their work.

**Figure 1:**
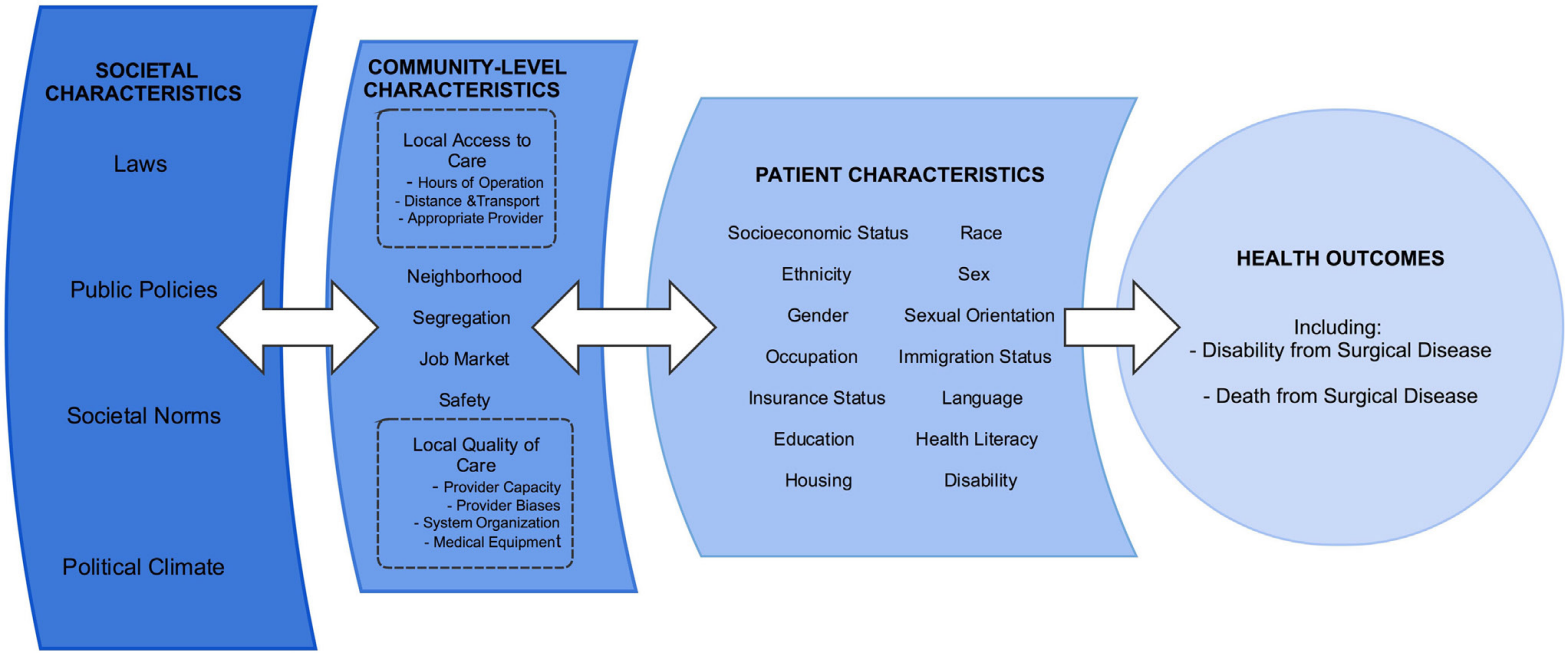
**Conceptual diagram of socially responsible surgery**.

The principles of SRS can be used to consolidate the efforts in research, education, and advocacy across the spectrum of care for the underserved. Figure 1 provides a conceptual diagram of the many factors involved in providing high quality surgical care to underserved communities. We acknowledge that many surgeons have long cared for these patients, largely without acclaim and often to the disadvantage of their comfort and compensation. Unifying the field under the SRS rubric may increase the academic rigor of these topics and facilitate support from the wider community of surgeons and public health practitioners.

This article highlights the current scope of practice within rural surgery, urban surgery, and global surgery and demonstrates the benefits of supporting the unifying concept of SRS. We searched PubMed using combinations of the MeSH terms “Vulnerable Populations,” “General Surgery,” “Healthcare disparities,” “Healthcare quality, access, and evaluation,” and “Outcome assessment (health care).” We additionally reviewed the reference lists of highly relevant articles. We examined the reports of the Lancet Commission on Global Surgery and the World Bank Disease Control Priorities, 3rd Edition: Essential Surgery. In addition, we summarize the work being done in SRS at our institution and conclude by offering next steps to help establish SRS as an important and impactful field within surgery and public health.

## Current Conditions

**Figure 2:**
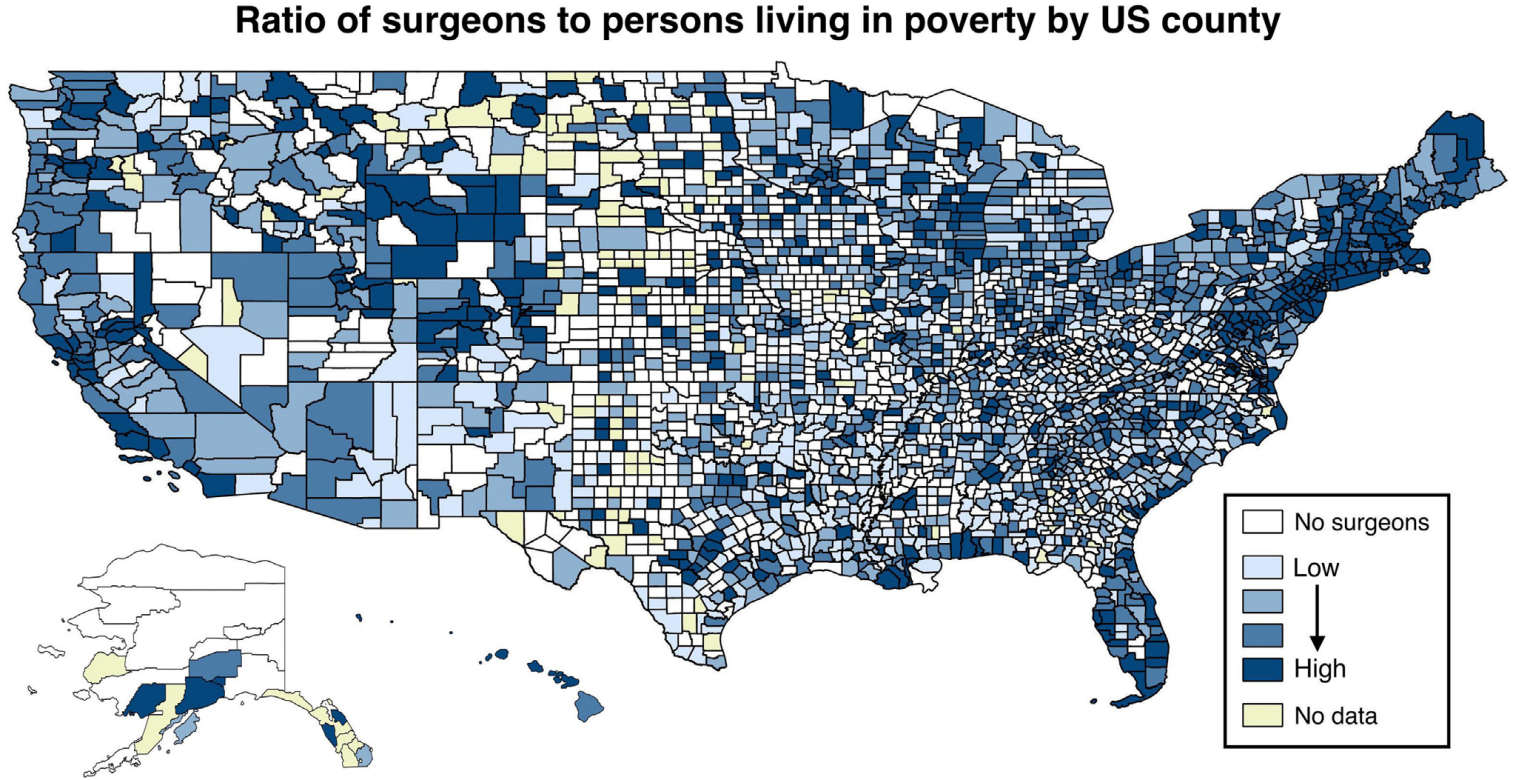
**Ratio of surgeons to persons living in poverty, by US county**. Plotted is the natural logarithm of the number of surgeons divided by the number of people living in poverty, by quartiles. Sources: AMA Masterfile (5); Centers for Disease Control and Prevention (6).

There is increasing recognition of surgery as an essential part of a robust health system, and yet access to surgical care remains scarce in resource-poor settings both domestically and globally (1–3). In the US, surgeons are more likely to practice in affluent urban and suburban areas, creating a workforce distribution that limits access for both rural and inner-city patients (4). Figure 2 presents a ratio of the number of surgeons to the total number of people living in poverty, by US county. As can be readily noted, there are many counties with no surgeons whatsoever. Of the 20 US counties with the lowest poverty rates (averaging 4.28%), 18 counties have surgeons, averaging 49.9 surgeons per 100,000 population. Of the 20 US counties with the highest poverty rates (averaging 43.5%), only 5 counties have any surgeons, averaging 4.7 surgeons per 100,000 population.

In both rural and urban US, underserved populations—the poor, the uninsured, the uneducated, the incarcerated, the homeless, racial minorities, and others—suffer higher rates of traumatic injury (4, 7), present with cancers at later stage (8), and receive worse surgical care (9–11). Globally, there is a dearth of surgical services available to the majority of the world’s population. An estimated 5 billion people worldwide lack access to surgical care (12, 13), with the poorest third of humanity receiving only 3.5% of the world’s operations (3, 14).

Wherever underserved populations live, there are deplorable disparities in health and, more concerning, disparities in health care. Surgeons have an integral role in reducing these disparities. The following sections will briefly explore the disparities in health, the disparities in health care, and describe the work currently being done in rural surgery, urban surgery, and global surgery.

### Surgical Needs and Access in Rural US

The burden of several surgical diseases is higher in rural settings than urban settings. For example, patients with abdominal aortic aneurysms living in rural areas have greater risk of rupture, are more likely to be transferred to another hospital for surgical repair, and less likely to undergo definitive repair (15). After controlling for other factors, patients in rural settings are less likely to have an appropriate bariatric surgery evaluation and treatment compared to those in urban areas (16). In addition, rural hospitals have significantly higher mortality rates among patients undergoing complex cancer surgery (17). And overall, individuals living in rural areas are also likely to be described by other social characteristics known to be associated with worse health outcomes, including minority status, low socioeconomic status, older age, public insurance, and greater comorbidity (16).

There is lack of access to surgical care in rural areas, as nearly one-third of all US counties, 95% of which were “non-metropolitan,” had no practicing surgeons (18). The lack of access to a local surgeon experienced by these 9.5 million Americans directly correlates with increased morbidity and mortality (4, 19).

Despite these challenges, many surgeons practicing in rural areas have created systems for excellent patient care and outcomes. Surgeons at a rural hospital in central Illinois provide primary care for their patients in addition to perioperative management and surgical treatment. Through this integrated care model, they achieve a surgical complication rate of only 4%, lower than in many urban areas (20). The authors suggest their integrated care model could be used to recruit medical students into surgical training and alleviate shortages of rural clinicians.

### Surgical Needs and Access in Urban US

The urban underserved also bear a high surgical burden when compared with other populations. Several studies have found that individuals living in urban areas had higher risk for late-stage presentation of breast, colorectal, lung, and prostate cancers, a pattern termed urban disadvantage (21–24). There is an alarming increase in fatal urban trauma (25), which is disproportionately suffered by young, impoverished black and Latino males (4, 26). Both minority race and low insurance status are strongly associated with worse outcomes following traumatic injury (11, 27). Uninsured trauma patients are twice as likely to die from their injuries as insured trauma patients. Black trauma patients are 20% more likely to die from their injuries as white patients (11).

Although many urban communities are located near major tertiary and academic medical centers, there is poor access to surgical services for the urban underserved. Urban Medicare beneficiaries are even less likely than rural Medicare beneficiaries to undergo surgical procedures (28). Despite protocols designed to standardize the care of injury at urban trauma centers, racial minorities receive worse initial management (10) and are less likely to be discharged to rehabilitation facilities (9). Urban safety-net hospitals provide essential services to underserved populations that face unique social, cultural, and linguistic barriers to care. However, these hospitals are insufficient in supply, and hospitals serving a high percentage of uninsured patients and ethnic minorities are disproportionately likely to close, decreasing access to care and resulting in worse health outcomes (29, 30).

Much of the current work to correct urban disparities involves injury prevention, including outreach programs at urban schools and community centers (31, 32). Community-based programs designed to reduce interpersonal violence have been successful (33). However, considerable work remains to improve surgical access for urban underserved populations. This will require concerted efforts at individual hospitals, as well as advocacy at state and federal levels.

### Surgical Needs and Access Globally

Surgical disease, including traumatic injury, is a leading cause of death and disability throughout the world and is a tremendous burden in low- and middle-income countries (LMICs) (34). Surgical disease has been estimated to cause 4.7 million deaths (10.4%) per year in LMICs and accounts for 14.2% of all disability-adjusted life years (3). Provision of 44 essential surgical procedures identified by The World Bank would avert 1.5 million deaths per year and are among the most cost-effective health interventions available to LMICs (35).

**Figure 3:**
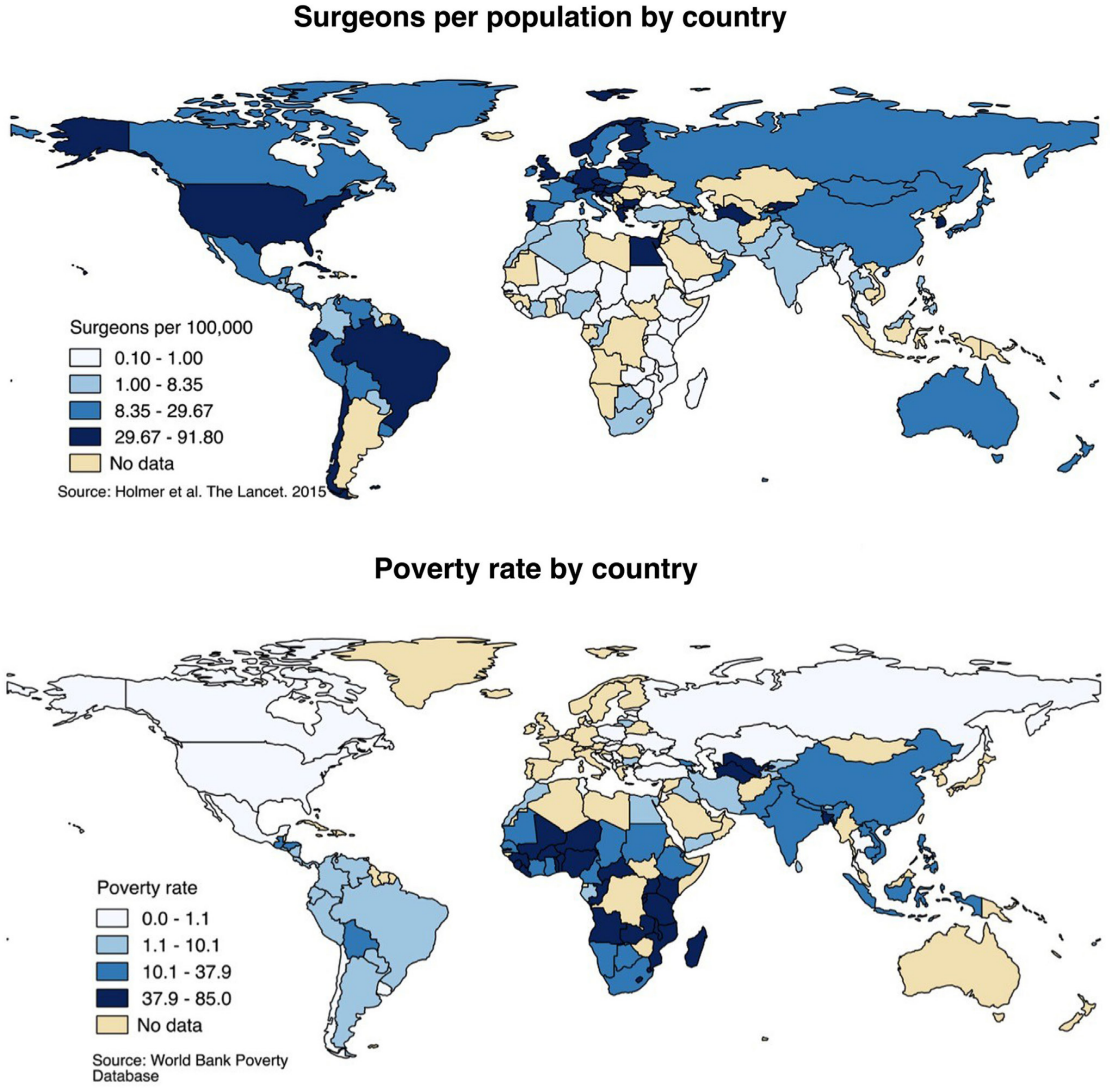
**Surgeon distribution and poverty rates, global**. Surgeon distribution reflects number of surgeons of any specialty per 100,000 population, by country and presented by quartile. Poverty rates reflect the percentage of the population living below the international poverty rate of living on less than $2 per day. Sources: Holmer et al. (36); World Bank Poverty Database (37).

Five billion people lack access to surgical care, and the vast majority live in rural areas of LMICs (12). There is substantial disparity in access to surgical care between LMICs and wealthy countries. Figure 3 presents surgeon density and poverty rates by country and demonstrates that people living in poor countries have much worse access to surgery than those living in high-income countries. On average, high-income countries have 14 operating theaters per 100,000 people compared to less than 2 operating theaters per 100,000 people in low-income countries (12). There is estimated to be only 1 African surgeon for every 100 American surgeons (1). Surgeons in LMICs are almost always located in the largest cities, leaving predominantly rural populations without any access to surgical care.

In the past decade, there has been significant scholarship and advocacy for improvements in global surgical care. The World Bank released a landmark volume, Disease Control Priorities, 3rd Edition: Essential Surgery. Similarly, The Lancet Commission on Global Surgery recently published its findings and recommendations. There is increasing recognition that surgery is a fundamental element of health care and a critical resource for health systems to address the global burden of disease. As governments grapple with how to best protect the health of their populations, surgeons need to advocate for emergency and essential surgery as a cost-effective means of preventing death and disability (38).

## Importance of Defining SRS

The burden of surgical disease and the challenges to management demonstrate that achieving optimal health outcomes requires more than excellent perioperative care. Surgeons can and should regularly address the social determinants of health experienced by their patients. Formalized research and training opportunities are needed to meet the growing enthusiasm among surgeons and trainees to develop their practice as socially responsible surgeons.

Unifying the work being done in rural, urban, and global surgery benefits surgeons working in these fields who typically share similar values, including a belief that all people have a right to high quality surgical care regardless of social status, and a commitment to fighting inequities that cause health-care disparities. These three spheres also face similar challenges, and solutions that improve surgical access and quality in one setting may be translatable to others. Research, education, and advocacy can be improved if surgeons doing similar work in different settings recognize their common goal of improving surgical care for underserved populations and disadvantaged patients.

Socially responsible surgery is an opportunity for surgeons to participate in public health and health system improvement, where their contributions have often been overshadowed. Despite the major recent accomplishments of describing the global burden of surgical disease and access to essential surgery, surgeons are still commonly perceived as focused on the narrow scope of operative treatment. Both policymakers and the broader public neglect to recognize surgeons as having an ongoing presence in the care of their patients and the health of communities. This is an oversight of the real contributions of many surgeons, and a misrepresentation of the role surgeons must assume in the care of the public’s health.

Practitioners and trainees looking for work within SRS often have difficulty finding support. Rural surgery and global surgery are gaining recognition as distinct pursuits, and a number of dedicated programs at academic medical centers now exist to support scholarship in each. Urban surgery is less well defined but is exemplified by the practice of surgeons caring for underserved populations within major cities. Awareness of these opportunities is low among residents and medical students, who commonly think of primary care specialists as the champions of socially responsible care. SRS may be a useful way to engage trainees interested in surgery who are also passionate about caring for underserved populations.

## Work in SRS at Our Institution

As surgeons and trainees in the Department of Surgery at Boston Medical Center (BMC) and the Boston University School of Medicine (BUSM), we have made significant progress in our work in SRS. There are several examples of programs at our institution addressing social determinants of health among our surgical patients, and exemplifying the practical application of SRS.

- The Community Violence Response Team (CVRT) is comprised of mental health professionals who provide counseling and community resources to and advocate for victims of violence and is led by a faculty member in the Department of Surgery. In partnership with the hospital-based Violence Intervention Program, the CVRT frequently consults with our hospital’s trauma surgeons on appropriate strategies and resources for prevention of future interpersonal violence. The team also educates trainees about injury prevention and breaking cycles of violence.
- The BMC Preventive Food Pantry provides access to adequate nutrition, which is an essential component of health and recovery from surgery. The Food Pantry provides a variety of foods, including fresh fruits and vegetables, to nearly 7,000 food insecure families every month without charge. Surgeons and trainees at BMC can refer patients to the food pantry for access to culturally appropriate and nutritious foods.
- The Boston Health Care for the Homeless Program’s Barbara McInnis House is a 104-bed medical respite facility that provides 24/7 medical support for patients lacking housing. The McInnis House cares for patients who are ready for hospital discharge but too infirm to return to shelters or the street. Medical care is coordinated between the BMC surgical team and McInnis House clinicians, with greatly improved postoperative outcomes in this vulnerable population.
- The Boston Medical Legal Partnership is one of the nation’s oldest and most renowned organizations providing underserved families with access to comprehensive legal services, helping them address issues related to housing, education and employment, income supports, legal status, and personal and family stability and safety.

In addition, the BUSM student Surgical Interest Group’s successful program matching medical students with residents and faculty by research interests has introduced an SRS research track highlighting and streamlining opportunities for research and training. The research track will incubate a community of surgeons and trainees at our hospital committed to addressing social barriers to surgical care. Our future goals include partnership with the Boston University School of Public Health to develop curricula addressing surgical disease at a community level.

## Next Steps

Fostering community around SRS will bring recognition and support for surgeons caring for underserved patients and enable surgeons to more effectively advocate for the resources needed to carry out work in all areas of SRS, including research, education, and advocacy. It is likely that there is already considerable work being done by surgeons and trainees at institutions around the country to address the social determinants of health affecting their patients. SRS provides an opportunity to recognize these ongoing efforts and to create a community of surgeon researchers, educators, and advocates.

As surgeons and health-care leaders, we have a responsibility to advocate for improving surgical care as one of the many needs and priorities within broader health-care systems. We must confront the disparities in health and in health care that exist within the spectrum of surgical disease and be committed to reducing these disparities to improve surgical access and outcomes for patients from underserved populations. Now is the ideal time to support the unifying concept of SRS.

## Author Contributions

TO, TR, NS, MF, DH, MJ, DK, and TD designed the study. TO, TR, and JM conducted the literature review. TO and TR prepared the manuscript. TO, TR, and TT prepared the figures. FS, DK, and RM edited the manuscript. All the authors approved the final version of the manuscript.

## Conflict of Interest Statement

The authors declare that the research was conducted in the absence of any commercial or financial relationships that could be construed as a potential conflict of interest.
